# Pan-American Notes

**Published:** 1901-08

**Authors:** 


### PAN-AMERICAN NOTES.

"FAIR JAPAN, one of the many interesting foreign countries on the Midway, was installed at the special instance of Director General Buchanan and every effort was made to secure a representative exhibit from the Japan Islands. Mr. Kushibiki, the manager of Fair Japan, was untiring in his efforts to build the village in a most attractive manner, and after everything was finished, the one difficulty was the procurement of the Geisha girls. They, however, have arrived at last and these young women are attractive, pretty and talented. Naome is a celebrated beauty of Tokio and will win her way into popular favor immediately. The young women have brought their native instruments and the merry tinkle of the samisen is now heard to the accompaniment of graceful tripping and gliding of the Geisha maidens.

Sadly enough M. Kushibiki, just as the Geishas arrived, sustained an injury necessitating amputation of a leg.

"Esau,"the chimpanzee, exhibited by Frank C. Bostock at the exposition, gave a special reception at the Iroquois Hotel, July 16, to forty or more prominent physicians. After the seance, the physicians examined "Esau," and pronounced him a most wonderful specimen.

Dr. William H. Heath, on invitation, gave a short clinical lecture on the resemblance between "Esau" and a human being. He said that the chimpanzee's head showed remarkable intelligence, and that his anatomical formation resembled closely that of a man of the lower order. Afterward several of the medical men made examinations of the "missing link."

The competition for the fireworks contract has closed and Pain will probably be the winner. The displays shown in competition were more brilliant than anything in the fireworks line which has ever been seen in Buffalo.

Bostock's animal show is drawing the crowds. The arena is the most instructive place on the Midway. Bostock has gathered together a most interesting collection of animals and they are shown in all degrees of training. A feature which has recently been added, and which is on the whole entirely novel and new, is the throwing open to the public of the training quarters where the animals in their wild state are put through their lessons for appearance in the big arena. Some of the wildest and most exciting battles between the trainers and animals that ever took place outside of the native wilds occur here.

The Midway will have a day of its own August 3, and it will be the biggest day of the whole exposition. The display will be bigger and better and more interesting than any special day which has been fixed upon and the events will follow one another without a break from early morning until long after midnight. It is expected that the illumination of the grounds will be continued all night and the Midway Day will not end until the rise of the sun on the next day. One of the features which has been decided upon is a mammoth dancing fete, in which will be seen all the dancers from all corners of the world who are at present performing in the different villages and streets on the Midway. They will be seen in one big display.

THE infant incubator at the junction of the Midway and the Mall has a constant stream of people going in and out. There are now 18 babies prematurely born in the incubators and the scientific rearing of these little human beings hanging between life and death by the slenderest thread, is most interesting not alone to scientists, but to the many mothers who go to the exposition. The incubator is in charge of physicians and the babies are cared for by nurses trained for this work alone. One can neglect seeing any other place on the grounds rather than this. It is not alone interesting; it is popularly instructive.

THUS far the Emergency Hospital has cared for over 3,000 patients. This number will be largely increased during the last three months of the exposition, for it is expected that the attendance will be more than four times the average of the first three months.

THE railroads have promised to make rates now and the crowds will begin to flock to Buffalo. Already some of the roads have made a cut and the number of people who visit the Rainbow City is appreciably increased.

DURING the extremely hot weather there were a great many cases of heat prostration, the highest number in one day being 38. There has not been received in the hospital thus far a case of sunstroke.

HARDLY a day passes that a dozen or so of women and children are not received in the Emergency Hospital as patients, suffering with a slight stroke of illness. Many a woman overcome by sudden indisposition has been taken to the hospital, where under the careful ministration of the very excellent nurses on duty they have soon recovered.

THE firework displays are features which draw night crowds. They keep people off the Midway, but furnish an entertainment of dazzling and gorgeous brilliancy.

DR. ROSWELL PARK'S excellent medical organisation has been warmly complimented by visiting physicians and sanitarians, who speak in the highest terms of the conduct of the hospital. Dr. Park's supervision extends over all the grounds and buildings, and not a day passes that the sanitary officer on his staff does not report to him exactly the sanitary condition of the exposition. Constant watchfulness keeps the foreigners clean, and surprise is expressed at the perfect sanitary supervision.

THE Crèche is now in operation at the exposition and babies may be checked with a certainty that they will receive the best possible care. The Crèche is a large tent, near the Emergency Hospital, equipped with every modern contrivance for attention to the infants.

THE African Village on the Midway is probably the most curious collection of foreigners at the Pan. They live in their village exactly as they do in their own country—half-naked. Their dances are weird, their music of the most primitive character. The most interesting feature of their methods is the way in which babies are carried across the mother's back.

PAINTED HORSE, a Pawnee chief, fell from the canvas cliff in front of the Indian Congress to the pointed rock ravine, 20 feet below and landed on his head. Old War Bonnet, the hereditary medicine chief of the tribe found Painted Horse unconscious and informed his tribe that their chief was dead. Then he proceeded to blow in his ears and nostrils, rubbed queer mixtures into his hair and lo, Painted Horse came to life. Then the tribe gave War Bonnet lots of presents because he had worked a miracle and brought the dead chief to life.

THE first death since the opening of the exposition occurred at the Indian Congress on the 13th of July, and was in a way accidental. John Ghost Dog, the year-old child of Ghost Dog and Plays-on--Flute, ate some partially cooked rice. The papoose choked and drew some of the hard grains of rice into its lungs. Almost immediately it developed a pneumonia and died within six hours. The child was buried at Pine Hill Cemetery. The ceremonies attending the funeral were unique. There was the usual four days of death, dancing and chanting of the famous spirit song of the Pawnees. Crumbs of bread were sprinkled in the blanket in which the little body was wrapped, to provide food for the spirit and the mysterious guide from the happy hunting grounds, which comes always to the dead to conduct the spirit to its new home.

The family mourning continues for a full month. Then the daily routine of life is resumed, the spirit by that time having reached its new abiding place.

CHIEF Little Wound, the old sachem, who attracted so much attention while he was at the Indian Congress, and who was sent home to the reservation at Pine Ridge, S.D., is dead. He went to the happy hunting grounds on the 18th inst., from his tepee at Pine Ridge.

THE hot weather made the Eskimos sigh for the frozen fields of their far away home. They dress in linen garments, except when they are giving exhibitions. One untutored son of the ice belt asked concessionaire Taber to please make a prayer for ice to come.

THE health of the foreigners is remarkably good. This is due to two causes. They are under constant supervision and they are compelled to keep clean.

THE action of the directors in making the Sunday admissions 25 cents, children 15 cents, was a very popular move, and with great propriety might have been taken in the beginning.

